# Molecular Docking and Molecular Dynamics Studies on Selective Synthesis of α-Amyrin and β-Amyrin by Oxidosqualene Cyclases from *Ilex Asprella*

**DOI:** 10.3390/ijms20143469

**Published:** 2019-07-15

**Authors:** Zhixue Wu, Hui Xu, Meiling Wang, Ruoting Zhan, Weiwen Chen, Ren Zhang, Zaoyuan Kuang, Fengxue Zhang, Kui Wang, Jiangyong Gu

**Affiliations:** 1 Research Centre for Integrative Medicine, Guangzhou University of Chinese Medicine, Guangzhou 510006, China; 2 The Second Clinical College, Guangzhou University of Chinese Medicine, Guangzhou 510006, China; 3 Research Centre of Chinese Herbal Resource Science and Engineering, Guangzhou University of Chinese Medicine, Key Laboratory of Chinese Medicinal Resource from Lingnan (Guangzhou University of Chinese Medicine), Ministry of Education, Joint Laboratory of National Engineering Research Centre for the Pharmaceutics of Traditional Chinese Medicines, Guangzhou 510006, China; 4 Department of Medical Biotechnology, College of Basic Medical Science, Guangzhou University of Chinese Medicine, Guangzhou 510006, China

**Keywords:** oxidosqualene cyclase, amyrin, selective synthesis, molecular modeling, residue-product interaction

## Abstract

Amyrins are the immediate precursors of many pharmaceutically important pentacyclic triterpenoids. Although various amyrin synthases have been identified, little is known about the relationship between protein structures and the constituent and content of the products. IaAS1 and IaAS2 identified from *Ilex asprella* in our previous work belong to multifunctional oxidosqualene cyclases and can produce α-amyrin and β-amyrin at different ratios. More than 80% of total production of IaAS1 is α-amyrin; while IaAS2 mainly produces β-amyrin with a yield of 95%. Here, we present a molecular modeling approach to explore the underlying mechanism for selective synthesis. The structures of IaAS1 and IaAS2 were constructed by homology modeling, and were evaluated by Ramachandran Plot and Verify 3D program. The enzyme-product conformations generated by molecular docking indicated that ASP484 residue plays an important role in the catalytic process; and TRP611 residue of IaAS2 had interaction with β-amyrin through π–σ interaction. MM/GBSA binding free energy calculations and free energy decomposition after 50 ns molecular dynamics simulations were performed. The binding affinity between the main product and corresponding enzyme was higher than that of the by-product. Conserved amino acid residues such as TRP257; TYR259; PHE47; TRP534; TRP612; and TYR728 for IaAS1 (TRP257; TYR259; PHE473; TRP533; TRP611; and TYR727 for IaAS2) had strong interactions with both products. GLN450 and LYS372 had negative contribution to binding affinity between α-amyrin or β-amyrin and IaAS1. LYS372 and ARG261 had strong repulsive effects for the binding of α-amyrin with IaAS2. The importance of Lys372 and TRP612 of IaAS1, and Lys372 and TRP611 of IaAS2, for synthesizing amyrins were confirmed by site-directed mutagenesis. The different patterns of residue–product interactions is the cause for the difference in the yields of two products.

## 1. Introduction

Oxidosqualene cyclases (OSCs) belong to a multi-gene family of enzymes that convert a linear molecule of 2,3-oxidosqualene (OS) to polycyclic products, such as phytosterols or triterpenoids [1,2,3]. Triterpenoids are an important class of natural products with wide distribution in animals, fungi, and plants [4,5,6]. These structurally diverse triterpenoids have anti-tumor, cognitive enhancement, anti-aging, anti-inflammatory, and hypoglycemic activities [7,8]. Enzymatic cyclization of squalene and oxidized squalene is the most significant step in sterol and triterpenoid biosynthesis [3]. OSCs are the only enzymes responsible for the entire process, but the cyclization mechanism remains unclear. Using OSC irreversible inhibitors and OSC mutation analysis, the highly conserved sequence of DCTAE was shown to be implicated in substrate binding [9,10]. The site-directed mutation of squalene-hopene cyclase (SHC) sequence DDTAVV motif to DCTAEA resulted in the conversion of the enzyme substrate from squalene to OS [11]. Through a series of protonation, cyclization, rearrangement, and deprotonation reactions of OS to form complex carbon skeletons, the entire enzymatic process follows the famous rules of bioisoprene [12].

More than 30 OSCs have been cloned and sequenced, including the synthetases of lanosterol, cycloartenol, β-amyrin, and lupinol [13,14]. Multifunctional OSCs which can produce more than one product have also been identified in many plants [15,16,17]. However, the yields for different products differ greatly. For example, two multifunctional OSCs separated from *Ilex asprella* in our previous work can simultaneously synthesize α-amyrin and β-amyrin, which can be further transferred into ursane and oleanane-type triterpenoids, respectively [16]. The two full-length cDNAs of OSCs were cloned and expressed in *Saccharomyces cerevisiae,* which were named IaAS1 and IaAS2. The products of IaAS1 and IaAS2 were identified and quantified by GC-MS. Both products were synthesized by IaAS1 and IaAS2. α-Amyrin was the main product of IaAS1, and the ratio of α-amyrin to β-amyrin was about 4:1. β-Amyrin was the main product of IaAS2, and the ratio of α-amyrin to β-amyrin was about 1:19 [16]. Several site-directed mutations of other OSCs (AsAS: β-amyrin synthase of *Avena strigose* [18], AtCYC: Cycloartenol synthase of *Arabidopsis thaliana* [19], AtCBS: Cucurbitadienol synthase of *Arabidopsis thaliana* [20], AtCPI: Cyclopropylsterol-cycloisomerase of *Arabidopsis thaliana* [21], AtLSSl: Lanosterol synthase of *Arabidopsis thaliana* [22], AtLUP1: Lupeol synthase 1 of *Arabidopsis thaliana* [18], CcLSS: Lanosterol cyclase of *Cephalosporium caerulens* [23], EtAS: β-amyrin synthase of *Euphorbia tirucalli* [24,25,26,27], OeLS: Lupeol synthase of *Olea europaea* [28], PgAS: β-amyrin Synthase of *Panax ginseng* [28], and ScLSS: Lanosterol cyclase of *Saccharomyces cerevisiae* [29,30,31,32,33,34,35,36,37]) have been performed (Supplementary Table S1). Multi-sequence alignment of sequences of IaAS1, IaAS2, human lanosterol synthase and seven enzymes from plants (AsAS, AtCYC, AtLSS1, AtLUP1, EtAS, O2LS, PgAS) provides key information for identifying the interface of enzyme-substrate/intermediate/product interaction (Supplementary Figure S1).

The sequence alignment of IaAS1 and IaAS2 revealed several amino acid (AA) residues around the active sites, which might be responsible for product specificity. The underlying mechanism of selective synthesis has not been fully elucidated. A significant cause is the lacking of known structures of OSCs. At present, only the structures of squalene-hopene cyclase from *Alicyclobacillus acidocaldarius* [38,39,40] and human lanosterol synthase [41] have been reported. Therefore, it’s possible to construct the structures of IaAS1 and IaAS2 by homology modeling. Then molecular docking and molecular dynamics studies on both enzymes with α-amyrin and β-amyrin could provide more information of their selective catalytic activities. Theoretically, both thermodynamic and kinetics factors can influence the yields of products. However, it’s difficult to calculate the activation energy since the structures of intermediates are unknown. In this work, two similar products are produced by the same enzyme from the same substrate. Therefore, we can assume that the activation energies and entropy changes for the two enzyme-catalyzed reactions are roughly the same. Therefore, the difference of binding affinity between each product and enzyme would be the most important factor for the different yield of this product. In order to explore the mechanism of selective synthesis of two OSCs, a molecular modeling approach by combining homology modeling, structure evaluation, molecular docking, molecular dynamics simulations, MM/GBSA binding free energy calculations, and free energy decomposition was adopted.

## 2. Results and Discussion

### 2.1. Sequence Analysis

The common template for IaAS1 and IaAS2 obtained from the online BLAST tool was human lanosterol synthase (PDB: 1W6K [41]). The template structure had a large binding pocket forits product, and there was a hydrogen bond between lanosterol and the ASP455 residue. The sequence similarity and identity between the template and IaAS1 were 58.3% and 35.8%, respectively. The sequence similarity and identity between the template and IaAS2 were 57.1% and 38.2%, respectively. Therefore, the structure of human lanosterol synthase can be used as a template to construct the 3D structures of IaAS1 and IaAS2 by homology modeling.

Multi-sequence alignment of the template, β-amyrin synthase of *Euphorbia tirucalli* L. (ETAS) [42], IaAS1 and IaAS2 showed that there were conserved AA residues, such as MET256, TRP257, CYS258, TYR259, CYS260, PHE412, GLY413, ASP484, CYS485, and THR564 (Table 1). The functions of the highly conserved residues (TRP257, TYR259, and PHE413) in ETAS were examined. The major catalysis-related function of TRP257 and TYR259 residues is to yield their π-electrons to the cationic intermediates to stabilize the intermediates [42]. It has been reported that the acidic carboxyl residue ASP484 acts as a proton donor to initiate the polycyclization reaction [41]. Both CYS485 and ASP484 carboxyl group can form hydrogen bonds, which would promote the continuation of the catalytic reaction.

### 2.2. Verification and Evaluation of the Modeling Structures

**Figure 1:**
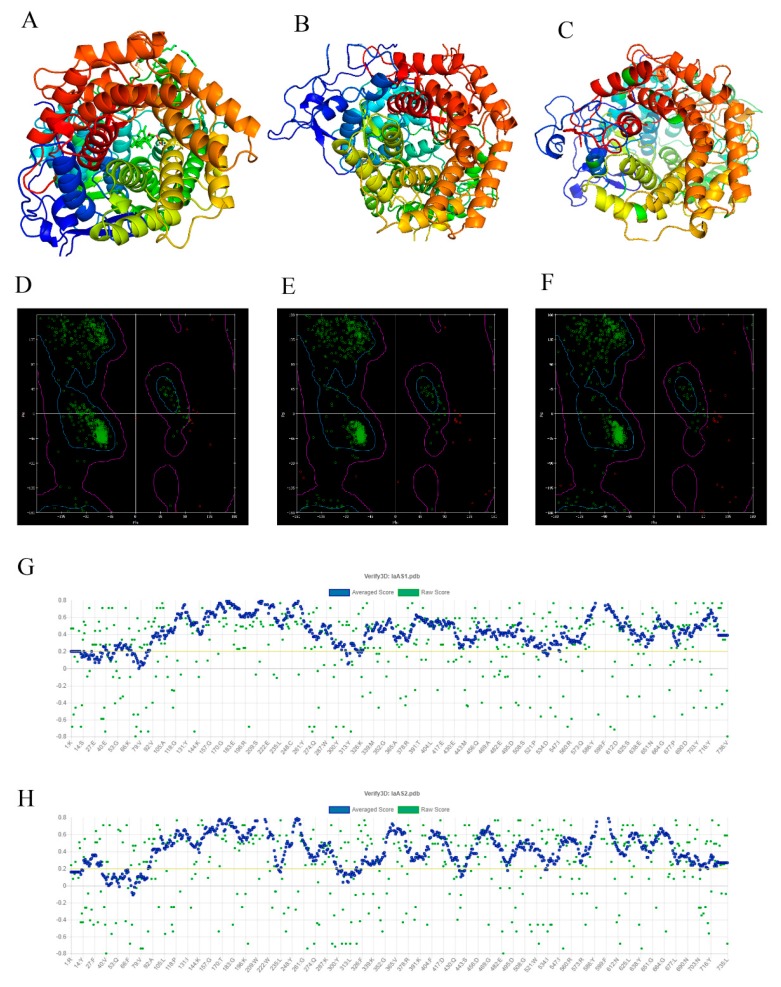
Homology modeling and evaluation of IaAS1 and IaAS2 structures. The Ribbon diagrams and Ramachandran plots for the template (**A**,**D**), IaAS1 (**B**,**E**) and IaAS2 (**C**,**F**), respectively. The 3D-1D compatibility of IaAS1 (**G**) and IaAS2 (**H**) structures evaluated by Verify 3D.

The MODELER program in Discovery Studio v2.5 (DS) was used to construct the structures of IaAS1 and IaAS2 (Figure 1A–C). Probability Density Function (PDF) Total Energy and Discrete Optimized Protein Energy (DOPE) Score of all output models were calculated by MODELER at the end of refinement. PDF *Total Energy* is the sum of the scoring function value of all homology-derived pseudo-energy terms and stereochemical pseudo-energy terms. The DOPE Score is an atomic distance-dependent statistical potential from a sample of native structures [43]. Smaller PDF *Total Energy* indicates that the model satisfies the homology restraints better. A lower DOPE Score also indicates a better model. The PDF *Total Energy* and DOPE Score for the best model of IaAS1 were 4255.02 and −92,571.68, respectively. The PDF *Total Energy* and DOPE Score for the best model of IaAS2 were 5400.99 and −93,962.45, respectively. The structures of IaAS1 and IaAS2 were further verified using Ramachandran plots and the online server of SAVES v5.0. The Ramachandran plots indicated that most regions for backbone dihedral angles ψ against φ of amino acid residues in both structures of IaAS1 and IaAS2 were energetically allowed (Figure 1D–F). The percentages of backbone dihedral angles ψ against φ in allowed region, marginal region, and disallowed region of IaAS1 and IaAS2 structures were 94.1, 5.3, 0.6; 93.2, 6.2, and 0.6, respectively. They were similar to the corresponding percentages of template structure (97.8, 2.2, and 0.0, respectively).

The Verify 3D program [44] determines the compatibility of a model (3D) with its amino acid sequence (1D) by assigning a structural class based on its location and environment (alpha, beta, loop, polar, nonpolar, etc.). The results showed that 91.58% of the residues had averaged 3D-1D score ≥0.2 for IaAS1, and the percentage of residues for IaAS2 was 85.85% (Figure 1G,H). The structural evaluation of MD optimization of the ERRAT program [45] on the online server of SAVES v5.0 showed that the overall quality factor of the model was 92.52% for IaAS1 and 92.51% for IaAS2. These results indicated that the modeling structures were reliable.

### 2.3. Molecular Docking

Molecular docking is a widely used structure-based drug design technique. It can predict the conformation of a ligand in the target binding site and calculate the binding energy [46]. Both α-amyrin and β-amyrin were docked with IaAS1 and IaAS2. The binding energy calculated by molecular docking between IaAS1 and α-amyrin or β-amyrin was −11.72 and −9.60 kcal/mol, respectively. The binding energy between IaAS2 and α-amyrin or β-amyrin was −5.71 and −12.25 kcal/mol, respectively. The binding affinity between the main product and the corresponding enzyme was higher than that of the by-product. Moreover, the order and difference of binding energies between the two products and the two enzymes calculated by molecular docking agreed with experimental results.

**Figure 2:**
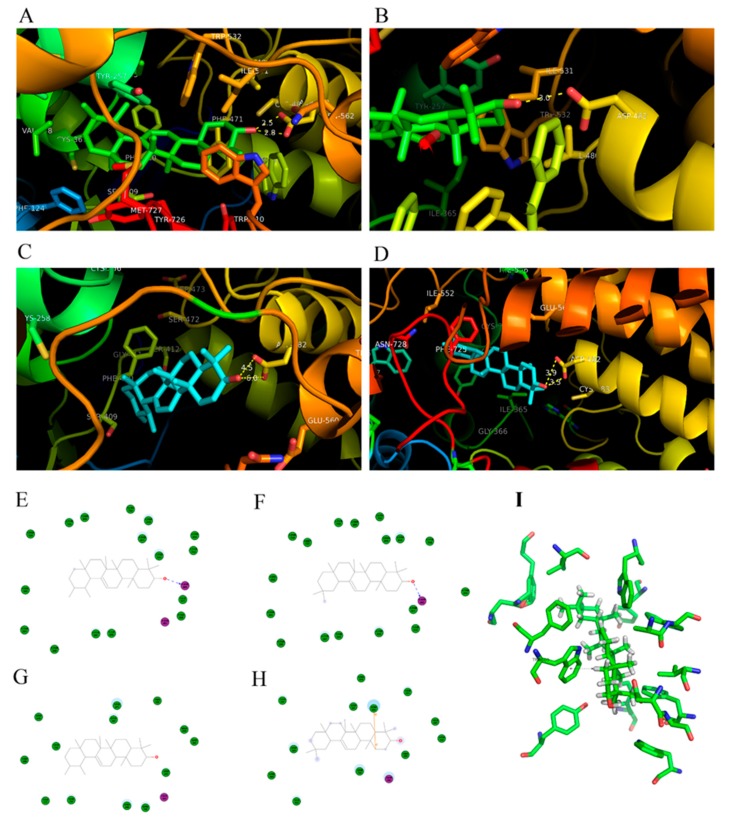
Molecular docking conformations and interactions between the enzyme and product for IaAS1_alpha (**A**,**E**), IaAS1_beta (**B**,**F**), IaAS2_alpha (**C**,**G**), and IaAS2_beta (**D**,**H**,**I**). The hydrogen bond donor and acceptor are colored as red and purple, respectively. Green and gray AA residues represent non-polar contact and any other contact, respectively. The first two AA residues were missing in the constructed structures; thus, the sequence number of AA residues is rearranged, i.e., ASP482 and TRP609 represent actual ASP484 and TRP611, respectively.

Figure 2A–D illustrated the docked conformations of α-amyrin (IaAS1_alpha) or β-amyrin (IaAS1_beta) in the binding site of IaAS1 or IaAS2. The ASP484 was a key AA residue that triggered the entire enzymatic reaction. The distance between the carboxyl of the ASP484 residue of IaAS1 and hydroxyl of α-amyrin (IaAS2_alpha) and β-amyrin (IaAS2_beta) was 2.5 Å and 3.0 Å, respectively. The distance between the carboxyl of the ASP484 residue of IaAS2 and hydroxyl of α-amyrin and β-amyrin was 4.5 Å and 3.3 Å, respectively. The interactions between enzymes and products were visualized by DS (Figure 2E–H). ASP484 played an important role in all four systems. Moreover, the TRP611 residue of IaAS2 had interaction with β-amyrin through π–σ interaction (Figure 2H,I). The different binding affinities between enzymes and products, and the distances between the ASP484 residue and hydroxyl of the product would be important reasons for the different ratio of the two products.

### 2.4. Molecular Dynamics Simulations and Calculation of Binding Free Energy

**Figure 3:**
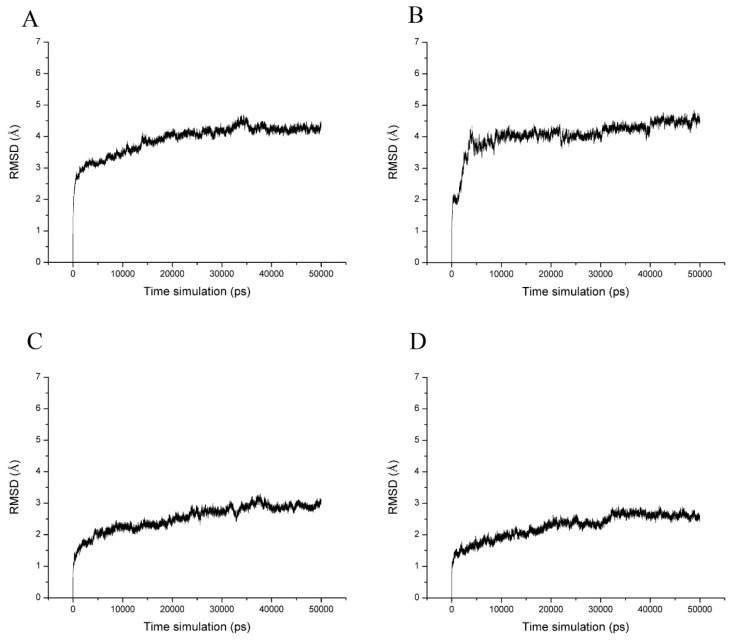
Time dependence of root mean square deviation (RMSD) of the backbone atoms (Ca, N, and C) for IaAS1_alpha (**A**), IaAS1_beta (**B**), IaAS2_alpha (**C**) and IaAS2_beta (**D**). The reference was the first snapshot of MD of each complex.

The four docked conformations were used as initial conformations of MD simulations. Each system was performed for 50 ns MD simulations. The structural stability of enzyme–product complex was evaluated by calculation of the root mean square deviation (RMSD). RMSD measures the deviation of a set of coordinates of a protein to a reference set of coordinates. The results showed that the complex of IaAS1 and α-amyrin or β-amyrin remained stable throughout the simulation. The complex of IaAS1 and α-amyrin or β-amyrin reached equilibrium after 35 ns (Figure 3). The average RMSDs of all atoms of enzyme-product complex after 35 ns for IaAS1_alpha, IaAS1_beta, IaAS2_alpha, and IaAS2_beta were 4.21 ± 0.13, 4.92 ± 0.19, 3.61 ± 0.19 and 3.34 ± 0.16 Å, respectively (Table 2). It indicated that all of the four complexes achieved equilibrium after 35 ns MD simulations. So, we chose the MD trajectories of the last 15 ns simulations for the following analysis.

The binding free energies for the four systems were calculated based on the MD simulations by MM/GBSA method. According to the RMSD curves, five stages of MD trajectories were selected to calculate the binding free energy (25–29 ns, 30–34 ns, 35–39 ns, 40–44 ns, and 45–49 ns). A total of 5000 snapshots for each section were used for calculation. MM/GBSA results showed that the average binding free energy of IaAS1 with α-amyrin and β-amyrin was −29.38 ± 1.02 kcal/mol and −25.10 ± 0.67 kcal/mol. The average binding free energy of IaAS2 with α-amyrin and β-amyrin was −21.95 ± 0.84 kcal/mol and −31.54 ± 1.18 kcal/mol (Table 2). The orders were well matched with the results of molecular docking. Moreover, the difference of binding free energy between IaAS1_alpha and IaAS1_beta was −4.28 Kcal/mol, while the difference of binding free energy between IaAS2_alpha and IaAS2_beta was 9.59 kcal/mol. It indicated two meanings. First, the binding affinity between α-amyrin and IaAS1 was higher than that between β-amyrin and IaAS1. So, the enzyme tended to produce more α-amyrin. But for IaAS2, the opposite was the case. Second, the α-amyrin to β-amyrin ratio catalyzed by IaAS1 would be lower than the ratio of β-amyrin to α-amyrin catalyzed by IaAS2. The MD simulation results were in good agreement with the experimental results.

### 2.5. Decomposition of Binding Free Energy

**Figure 4:**
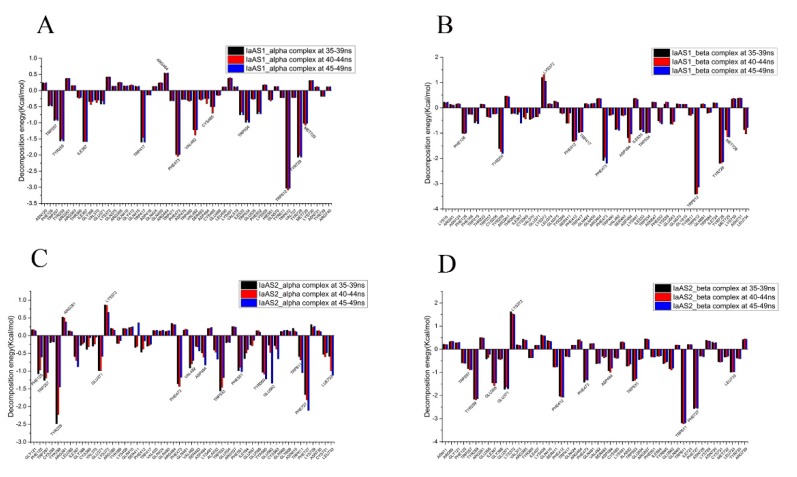
Decomposition of binding free energy of each residue for IaAS1_alpha (**A**), IaAS1_beta (**B**), IaAS2_alpha (**C**), and IaAS2_beta (**D**).

In order to further elucidate key AA residues which had more influence on binding free energy, per-residue decompositions were performed to generate the residue–product interaction spectra. According to the RMSD results, we selected three sections of MD trajectories (35–39 ns, 40–44 ns, and 45–49 ns) to decompose the binding free energy (Figure 4). Based on the different contributions to the binding free energy, the significance for binding affinity of each AA residue was determined. An AA residue may have a positive or negative contribution. The more negative the decomposed binding free energy of an AA residue, the more contributions to binding affinity this AA residue will have. Conversely, the more positive the decomposed binding free energy of an AA residue, the more repulsive effects the residue will have, which is not helpful to the catalytic activity of the enzyme.

Conserved AA residues such as TRP257, TYR259, PHE473, TRP534, TRP612, and TYR728 of IaAS1 (the conserved AA residues of IaAS2 were TRP257, TYR259, PHE473, TRP533, TRP611, and TYR727) had strong interactions with the corresponding product in all four complexes. Therefore, these AA residues would play important roles in the catalytic process.

The important AA residues for each system were ranked by the average contributions to the binding free energy for three sections of MD trajectories. For the IaAS1_alpha complex, TRP612 (−3.05 ± 0.03 kcal/mol, the unit was the same for following AA residues), TYR728 (−2.05 ± 0.03), PHE473 (−2.01 ± 0.03), ILE367 (−1.58 ± 0.01), TRP417 (−1.55 ± 0.08), TYR259 (−1.55 ± 0.03), VAL482 (−1.28 ± 0.09), MET729 (−1.04 ± 0.03), TRP534 (−0.96 ± 0.05), and TRP257 (−0.92 ± 0.02) were the top ten AA residues which can form strong interaction with α-amyrin (Figure 4A). The top ten AA residues for IaAS1_beta complex (Figure 4B) were TRP612 (−3.32 ± 0.16), TYR728 (−2.17 ± 0.03), PHE473 (−2.08 ± 0.12), TYR259 (−1.72 ± 0.08), PHE412 (−1.31 ± 0.02), ASP484 (−1.20 ± 0.17), MET729 (−1.06 ± 0.16), PHE126 (−1.01 ± 0.01), TRP534 (−0.99 ± 0.02), and TRP417 (−0.96 ± 0.01). TRP612, TYR728, and PHE473 contributed most, suggesting that they would play a key role in the synthesis of α-amyrin and β-amyrin. However, GLN450(0.54 ± 0.01) and LYS372 (1.19 ± 0.13) had negative contribution to binding affinity between α-amyrin or β-amyrin and IaAS1, respectively, which can make up a difference in the yield. Eleven important AA residues (TRP257, TYR259, PHE412, PHE473, VAL482, ASP484, TRP534, TRP612, MET729, ASN731, and LEU734) of IaAS1 are consistent with the results of site-directed mutations (Supplementary Table S1).

In the IaAS2_alpha complex, TYR259 (−2.05 ± 0.44), PHE727 (−1.86 ± 0.23), TRP533 (−1.40 ± 0.20), PHE473 (−1.33 ± 0.13), TRP257 (−1.17 ± 0.11), TYR560 (−1.12 ± 0.10), PHE551 (−0.97 ± 0.06), LEU733 (−0.90 ± 0.28), PHE125 (−0.89 ± 0.25), and GLU371 (−0.86 ± 0.23) contributed more to the binding free energy (Figure 4C). The key AA residues for IaAS2_beta complex (Figure 4D) were TRP611 (−3.20 ± 0.02), PHE727 (−2.55 ± 0.02), TYR259 (−2.16 ± 0.02), PHE412 (−2.06 ± 0.02), GLU371 (−1.69 ± 0.05), ILE367 (−1.49 ± 0.07), PHE473 (−1.36 ± 0.06), TRP533 (−1.33 ± 0.05), LEU733 (−0.98 ± 0.01), ASP484 (−0.92 ± 0.09), and TRP257 (−0.87 ± 0.02). Several AA residues such as LYS372 (0.79 ± 0.12) and ARG261 (0.47 ± 0.07) had strong repulsive effects for the binding of α-amyrin with IaAS2. But there was only one AA residue (LYS372, 1.55 ± 0.06) which had strong repulsive effects for the IaAS2_beta complex. Compared with the decomposed binding energy of TRP611 (−0.78 ± 0.24) in the IaAS2_alpha complex, the interaction between TRP611 and β-amyrin contributed much more in the IaAS2_beta complex. The above differences would be key factors for the difference in binding affinity between IaAS2 and the two products. Thirteen AA residues (TRP257, TYR259, PHE412, PHE473, VAL482, ASP484, TRP533, CYS563, TRP611, ASN728, ASN730, LEU733, and TYR735) of IaAS2 are consistent with the results of site-directed mutations (Supplementary Figure S1).

### 2.6. Quantification of Products Generated by Mutated IaAS1 and IaAS2

Trp612 was the most important AA residue for the binding of α/β-amyrin to IaAS1, while Lys372 was not conducive to the binding of β-amyrin to this enzyme. Regarding IaAS1, similar situations occurred for Trp611 and Lys372 (Figure 4). Moreover, polar solvation and van der Waals interactions contributed the most for Lys372 and Trp612/611 to the binding between product and enzyme, respectively. Therefore, to further confirm the importance of key AA residues for selective synthesis of both amyrins by IaAS1 and IaAS2, lysine and tryptophan in the two positions mentioned above were mutated to glycine and phenylalanine by using the technique of site-directed mutagenesis, respectively.

**Figure 5:**
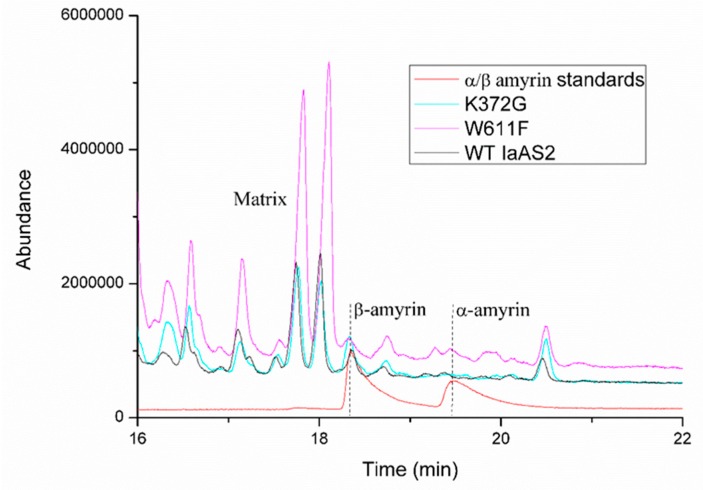
GC analysis of the products of mutated and wild type IaAS2.

Mutating the Lys372 to glycine and the Trp612 to phenylalanine abolished the catalytic ability of IaAS1; none of α-amyrin or β-amyrin was detected in the metabolite. It demonstrated that Lys372 and Trp612 were essential for the biosynthesis of amyrins. While for IaAS2, Lys372G and TRP611F mutants had a different impact on the biosynthesis of α/β-amyrin (Table 3 and Figure 5). Both mutants decreased the β: α products ratio, which meant that the selectivity of IaAS2 was weakened. The Lys372G mutant produced more α-amyrins than that of the TRP611F mutant. These results support our perspectives on the importance of residues involved in product binding affinity.

## 3. Materials and Methods

### 3.1. Homology Modeling to Construct Structures of IaAS1 and IaAS2

The sequences of the two amyrin synthases (IaAS1 and IaAS2) were retrieved from the PubMed. There were 762 AA residues in IaAS1 (GenBank: AIS39793.1) and 761 AA residues in IaAS2 (GenBank: AIS39794.1). The NCBI online Standard Protein BLAST tool (https://www.ncbi.nlm.nih.gov) was used to search template structure (PDB ID:1w6k [41]) for homology modeling. Sequence alignment by the Align Sequence to Templates module in Discovery Studio v2.5 (DS) was used to investigate highly conserved residues with following parameters: K-Tuple, Gap Penalty, and Top Diagonals were set to 1, 5, and 3, respectively; the multiple alignment scoring matrix was BLOSUM62; Gap Open Penalty, Multiple Alignment Gap Extension Penalty, Delay Divergent and Gap Separation Distance were set to 10.0, 0.05, 40.0, and 8, respectively; the rest were the default settings. The Build Homology Model module in DS was used to build models according to standard protocol. The optimization level was set to high, and 5 models were generated. The most reliable model was selected based on the least Discrete Optimized Protein Energy (DOPE) score and PDF Total Energy. The resulting model was evaluated by the Ramachandran Plot in DS and the online server of SAVES v5.0 (http://servicesn.mbi.ucla.edu/SAVES).

### 3.2. Molecular Docking

The AutoDock v4.2.6 and AutoDocktools v1.5.6 (ADT) [47] were used to calculate the best binding conformation of each product in the binding site of IaAS1 andIaAS2. The first step was to prepare the receptor structure of IaAS1 and IaAS2 by adding polar hydrogen atoms, a computing gasteiger charge and assigning the AD4 atom type in ADT. The following residues—TRP255, CYS256, TYR257, CYS258, VAL368, SER409, PHE471, ASP482, GLU486, TRP610, TYR726, MET727, PHE123, PHE166, PHE124, PHE550, THR484, GLU486, PHE410, GLY411, TRP217, CYS483 of IaAS1; TRP255, CYS256, TYR257, CYS258, SER409, PHE410, GLY411, PHE471, ASP482, CYS483, THR484, TRP609, PHE165, PHE549, PHE725, PHE619 of IaAS2—were assigned as flexible residues. Second, the structures of products were also prepared in ADT. Third, the energy grid maps were calculated by the AutoGrid program with the following parameters: The box-size was set to enveloping ligands and centered on the ligand; the grid spacing was set to 0.375 Å. Finally, the docking was carried out by the AutoDock program. The Lamarck’s genetic algorithm (LGA) was used to optimize the conformation of α-amyrin or β-amyrin in the binding pocket. The parameters for LGA were as follows: The number of individuals in population, the maximum number of energy evaluations, the maximum number of generations, and the rate of gene mutation were set as 150, 1.75 × 10^6^, 2.7× 10^5^, and 0.02, respectively. Other parameters were set to default.

### 3.3. Molecular Dynamics Simulations

The docking conformation with lowest binding energy was selected as the initial conformation for MD simulation. Gaussian 09 and antechamber programs were used to create the force field parameters for α-amyrin and β-amyrin. The ff14sb protein field [48] was used for the tleap program in AMBER 16 [49]. All the systems were immersed in a 15 × 15 × 15 Å cubic box with TIP3P water molecules. Na^+^ ions were used to neutralize the solution. The fully solvated system was then minimized in 3 stages by the sander program. The first stage involved the minimization of 2000 steps of steepest descent followed by 2000 steps of conjugate gradient with the constraining of all atoms of protein and ligand. In the second stage, only the atoms of backbone were constrained to minimize water and the side chains of protein by using the steepest descent minimization of 5000 steps followed by a conjugate gradient minimization of 5000 steps. In the third stage, the entire system was optimized without any constraint and the method was the same as with the second stage. The system was then heated gradually from 0 to 310 K in the NVT ensemble and equilibrated at 310 K for another 20 ps. Then 50 ns MD simulations were performed by the pmemd program. The coordinates were saved every 10 ps for subsequent analysis. The cpptraj program was used to calculate the root mean square deviation (RMSD).

### 3.4. Calculation and Decomposition of Free Energy

It was reported that the Molecular Mechanics/Generalized Born Surface Area (MM/GBSA) showed better performance in ranking the binding affinities for systems without metals than the Molecular Mechanics/Poisson Boltzmann Surface Area (MM/PBSA) [50,51,52]. Therefore, the binding free energy (ΔG_bind_) for the four systems was calculated using MM/GBSA methods by the MMPBSA.py program [53] in AMBER 16. The MM/GBSA method includes the calculation of the Van der Waals interaction energy, the electrostatic energy, the non-polar solvation free energy, and the polar solvation free energy. The contribution of binding energy of each residue was further decomposed into four parts: Van der Waals energy, electrostatic interaction, polar solvation energy, and non-polar solvation energy [54,55,56]. Therefore, this method can discover the key residues which are responsible for the different binding affinities of the two products.

### 3.5. Site-Directed Mutagenesis

In order to verify the credibility of the molecular docking and molecular dynamics modeling results, site-directed mutagenesis was carried out for IaAS1 and IaAS2, respectively. Two single mutants for IaAS1 (K372G or W612F) and IaAS2 (K372G or W611F) were obtained via inverse PCR (iPCR) [57]. Briefly, primers (Table 3) carrying the desired mutations for iPCR were phosphorylated by T4 polynucleotide kinase (NEB, Ipswich, MA, USA). Then, whole plasmid amplifications were performed via the phosphorylated primer pairs, using the pEXPR-IaAS1 or pEXPR-IaAS2 plasmid as a template. The resulting PCR products were purified, treated by Dpn I restriction enzyme for the digestion of template plasmids and cyclized using T4 ligase (NEB, Ipswich, MA, USA). The products were transformed into chemically competent Escherichia coli DH5α (TSINGKE, Guangzhou, China). The plasmids carrying the desired mutant genes were verified by sequencing. pEXPR-IaAS1, pEXPR-IaAS2, and their mutant plasmids were transformed into Saccharomyces cerevisiae WAT11 using the LiAc/SS carrier DNA/PEG method [58]. Transformants were individually cultured in SC-U medium (synthetic complete medium without uracil) (FunGenome, Beijing, China) containing 2% glucose (SC-U/Glu) and induced in 30 mL SC-U medium containing 2% galactose (SC-U/Gal). Metabolites extraction and GC-MS analysis were performed by following the methods described in our previous publication [16].

## 4. Conclusions

Both α-amyrin and β-amyrin can be synthesized by two multifunctional OSCs, but the yields for the two products differ greatly. The ratios of α-amyrin to β-amyrin produced by IaAS1 and IaAS2 are 4:1 and 1:19, respectively. In this work, a molecular modeling approach by combining homology modeling, structure evaluation, molecular docking, molecular dynamics simulations, MM/GBSA binding free energy calculations, and free energy decomposition was adopted to explore the mechanism of selective synthesis of amyrins by two oxidosqualene cyclases of *Ilex asprella*. On the basis of molecular docking conformations and visualized enzyme-product interactions, ASP484 was found to play an important role in the catalytic process, and TRP611 of IaAS2 had interaction with β-amyrin through π–σ interaction. The binding free energies predicted by molecular docking were consistent with the experimental results. Molecular dynamics simulations and MM/GBSA binding free energy calculations further strengthened the evidence. The results of binding free energy decomposition demonstrated that conserved AA residues such as TRP257, TYR259, PHE473, TRP534, TRP612, and TYR728 for IaAS1 (TRP257, TYR259, PHE473, TRP533, TRP611, and TYR727 for IaAS2) had strong interactions with the corresponding product in all four complexes. Residues would have negative contributions to binding affinity between the enzymes and products. Eleven AA residues of IaAS1 and thirteen AA residues of IaAS2 which have been identified as important AA residues are consistent with the results of site-directed mutations. These two enzymes also have uniquely important residues, such as ILE367, TRP417, TYR728 for IaAS1 and GLU358, GLU371, PHE727 for IaAS2. The different patterns of residue–product interactions play an important role in the enzymatic cyclization mechanism and are responsible for product determination. The importance of Lys372 and TRP612 of IaAS1, Lys372 and TRP611 of IaAS2 for synthesizing amyrins was confirmed by site-directed mutagenesis. The results give an insight into the mechanism that OSCs convert a single substrate into different products.

## Figures and Tables

**Table 1:** Conserved residues in template, ETAS, IaAS1 and IaAS2 sequences.

Enzyme	Conserved Residues
1W6K	LEU229 TRP230 CYS231 HIS232 CYS233 ASN382 GLY383 ASP455 CYS456 THR534
ETAS	MET256 TRP257 CYS258 TYR259 CYS260 PHE413 GLY414 ASP485 CYS486 THR565
IaAS1	MET256 TRP257 CYS258 TYR259 CYS260 PHE412 GLY413 ASP484 CYS485 THR564
IaAS2	MET256 TRP257 CYS258 TYR259 CYS260 PHE412 GLY413 ASP484 CYS485 THR564

**Table 2:** Predicted binding free energy by molecular dynamics (MD) simulations.

Complex	MM/GBSA ΔG_GBTOT_ (kcal/moL)						RMSD (mean ± SD)
	25–29 ns	30–34 ns	35–39 ns	40–44 ns	45–49 ns	(mean ± SD)	
IaAS1_alpha	−29.65	−30.26	−30.25	−28.87	−27.86	−29.38 ± 1.02	4.21 ± 0.13
IaAS1_beta	−26.06	−25.44	−24.40	−25.01	−24.60	−25.10 ± 0.67	4.92 ± 0.19
IaAS2_alpha	−21.29	−21.74	−21.14	−22.41	−23.16	−21.95 ± 0.84	3.61 ± 0.19
IaAS2_bet	−32.54	−32.86	−31.33	−30.98	−29.96	−31.54 ± 1.18	3.34 ± 0.16

**Table 3:** Primers used for IaAS1 and IaAS2 mutation.

Enzyme	Mutant	Primer	Sequence ^a^ (5′-3′)	Products Ratio
IaAS1	K372G	K372G-F	GGGAGTTTGCAAATGATGT	No products
		K372G-R	TTCTACACATCCTATAGTAATGTATCTGCTCT	
	W612F	W612F-F	TTCGGAATTTGCTTCCTCTATG	No products
		W612F-R	ATAACCATACCATGAACCATCAGG	
IaAS2	K372G	K372G-F	GGGGTACTATGTATGCTTGCTTG	β:α = 2.2:1
		K372G-R	TTCCACACATCCGATGGTG	
	W611F	W611F-F	TTCGGTGTGTGTTTCACATATG	β:α = 4.0:1
		W611F-R	GTTTCCATACCATGAACCATCAGAC	

Note: ^a^ Sequences underlined denote the codons for introduced amino acids.

## Supplementary Materials

Supplementary materials can be found at https://www.mdpi.com/1422-0067/20/14/3469/s1.

## Abbreviations

AA	amino acid
ADT	AutoDocktools v1.5.6
DOPE	Discrete Optimized Protein Energy
DS	Discovery Studio v2.5
IaAS1	amyrin synthase 1 of *Ilex asprella*
IaAS1_alpha	complex of α-amyrin and IaAS1
IaAS1_beta	complex of β-amyrin and IaAS1
IaAS2	amyrin synthase 2 of *Ilex asprella*
IaAS2_alpha	complex of α-amyrin and IaAS2
IaAS2_beta	complex of β-amyrin and IaAS2
iPCR	inverse PCR
LGA	Lamarck’s genetic algorithm
MD	molecular dynamics
MM/GBSA	Mechanics/Generalized Born Surface Area
MM/PBSA	Molecular Mechanics/Poisson Boltzmann Surface Area
OSC	Oxidosqualene cyclase
OS	2,3-oxidosqualene
PDF	Probability density function
SC-U	synthetic complete medium without uracil
RMSD	root mean square deviation
SHC	squalene-hopene cyclase
