# Supplementary material for: Molecular Docking and Molecular Dynamics Studies on Selective Synthesis of α-Amyrin and β-Amyrin by Oxidosqualene Cyclases from Ilex Asprella
Source: Int J Mol Sci. 2019 Jul 15;20(14):3469. doi: 10.3390/ijms20143469 (PMC6678101; doi:10.3390/ijms20143469)
Supplement: Supplementary file 1 [file ijms-20-03469-s001.pdf]

Supplementary Table S1. Mutagenesis studies of OSCs.

Enzyme	Mutation site	Function Changed
**AsAS**	S728F [1]	Convertes AsAS into an enzyme that makes tetracyclic (dammarane) instead of pentacyclic products.
**AtCAS1**	Y410C, H477Y, A469V, I481T, Y532H [2]	Alters product specificity: Y410C and H477Y mutants produce lanosterol as the dominant product, whereas the A469V, I481T and Y532H mutants produce a mixture of lanosterol and achilleol A.
**AtCYC**	Y410T [3]	Converts cycloartenol synthase to an oxidosqualene cyclase that forms lanosterol as its major product
**AtCYC**	Y118L [4]	Produces of cucurbitadienol as a major product
**AtCYC**	Y410T, H477N, I481V [5]	alters the position or electronic properties of the tyrosine by interaction with the phenolic hydroxyl group
**AtCBS**	L125Y [4]	Produces of parkeol
**AtCPI**	W112L [6]	Extends substrate specificity
**AtLSSl**	N477H [7]	Promotes the biosynthesis of lanosterol (88%) and parkeol (12%) by relocating the polar His257 and Tyr410 residues close to C-9 and C-ll.
**AtLSSl**	V481I [7]	Facilitates lanosterol production (25% lanosterol, 21% parkeol, and 54% cycloartenol) by enlarging the active site and reducing steric control.
**AtLUP1**	T729F [1]	Similarly resulted with AsAS.
**CcLSS**	H234E, H234Y, H234F, D456E, D456N, D456H [8]	Inactive
**EtAS**	V483, W534, M729 [9]	The Gly and Ala variants with a smaller bulk size at position 483 predominantly afford monocyclic camelliol C.<br>The Trp534 residue, with the largest steric bulk among all natural amino acids, is essential for high enzymatic activity. Various variants of Trp534 exhibit significantly decreased enzymatic activities and provide no aberrantly cyclized products.<br>Altering the steric bulk at the Met729 position affords the pentacyclic skeletons.
**EtAS**	F474 [10]	The Gly and Ala mutants produce significantly larger amounts of the bicyclic products and a decreased amount of beta-amyrin, indicating that the F474 residue was located near the B-ring formation site.
**EtAS**	D485N, C564A [11]	No activity of the D485N variant and significantly decreased activity of the C564A variant were found.
**EtAS**	W612, L734, Y736 [12]	The aliphatic variants (Ala, Val, Met) of Trp612 show almost no activity, but the aromatic variants exhibit high activities.<br>The L734G and L734A variants exhibit significantly decreased activities but yielded taraxerol in a high production ratio. The Val, Ile, and Met variants show markedly high activities (56-78% of wild-type activity).<br>The aliphatic variants of Tyr736 show markedly decreased activities, but the Phe mutant exhibit high activity (67%), which indicates that the electrons are critical for catalysis.
**OeLS**	L256W [13]	Produces exclusively beta-amyrin with only minor amount of lupeol.
**PgAS**	W259L [13]	Produces lupeol as a major product together with beta-amyrin in 2:1 ratio.
**PgAS**	Y261H [13]	Produces dammara-18,21-dien-3beta-ol (as a 3:5 mixture of E/Z isomer) together with minor amount of dammara-18(28),21-dien-3beta-ol.
**ScLSS**	T384Y, V454I [14]	Alters the product structure
**ScLSS**	H234X [15]	Changes in product specificity from lanosterol formation to either protosta-12,24-dien-3beta-ol or parkeol production
**ScLSS**	F699T [16]	Produces novel protosta-13(17),24-dien-3beta-ol as the sole truncated rearrangement product

**ScLSS**	T384Y, Q450H, V454I [17]	Produces parkeol but not lanosterol as the sole end product
**ScLSS**	I705X [18]	Affects lanosterol's C/D ring stabilization including 6-6-5 tricyclic and protosteryl C-17 cations and 17α/β-exocyclic side chain stereochemistry
**ScLSS**	Y99X [19]	Two truncated intermediates were isolated from the Y99X mutants.
**ScLSS**	Y510W/K [20]	Fails to complement cyclase-deficiency
**ScLSS**	C703I/H [21]	Generates an iridal-type triterpenoid
**ScLSS**	C457G, T509G [22]	Generates achilleol A as the major product

Note: Abbreviation of enzyme: AsAs, beta-amyrin synthase of *Avena strigosa*; AtCYC, Cycloartenol synthase of *Arabidopsis thaliana*; AtCBS, Cucurbitadienol synthase of *Arabidopsis thaliana*; AtCPI, Cyclopropylsterol-cycloisomerase of *Arabidopsis thaliana*; AtLSS1, Lanosterol synthase of *Arabidopsis thaliana*; AtLUP1, Lupeol synthase 1 of *Arabidopsis thaliana*; CcLSS, Lanosterol cyclase of *Cephalosporium caerulens*; EtAS, beta-amyrin synthase of *Euphorbia tirucalli*; OeLS, Lupeol synthase of *Olea europaea*; PgAS, beta-Amyrin Synthase of *Panax ginseng*; ScLSS, Lanosterol cyclase of *Saccharomyces cerevisiae*.

References

1. Salmon, M.; Thimmappa, R.B.; Minto, R.E.; Melton, R.E.; Hughes, R.K.; O'Maille, P.E.; Hemmings, A.M.; Osbourn, A. A conserved amino acid residue critical for product and substrate specificity in plant triterpene synthases. *Proc. Natl. Acad. Sci. USA.* **2016,** 113, 4407–4414.
2. Wu, T.K.; Griffin, J.H. Conversion of a plant oxidosqualene-cycloartenol synthase to an oxidosqualene-lanosterol cyclase by random mutagenesis. *Biochemistry* **2002,** 41, 8238–8244.
3. Herrera, J.B.R.; Wilson, W.K.; Matsuda, S.P.T. A tyrosine-to-threonine mutation converts cycloartenol synthase to an oxidosqualene cyclase that forms lanosterol as its major product. *J. Am. Chem. Soc.* **2000,** 122, 6765–6766.
4. Takase, S.; Saga, Y.; Kurihara, N.; Naraki, S.; Kuze, K.; Nakata, G.; Araki, T.; Kushiro, T. Control of the 1,2-rearrangement process by oxidosqualene cyclases during triterpene biosynthesis. *Org. Biomol. Chem.* **2015,** 13, 7331–7336.
5. Lodeiro, S.; Segura, M.J.; Stahl, M.; Schulz-Gasch, T.; Matsuda, S.P. Oxidosqualene cyclase second-sphere residues profoundly influence the product profile. *ChemBioChem* **2004,** 5, 1581–1585.
6. Rahier, A.; Karst, F. Plant cyclopropylsterol-cycloisomerase: key amino acids affecting activity and substrate specificity. *Biochem. J.* **2014,** 459, 289–299.
7. Kolesnikova, M. Investigation of triterpene biosynthesis in Arabidopsis thaliana. Doctoral thesis, Rice University, Texas, USA, 2008.
8. Abe, I.; Naito, K.; Takagi, Y.; Noguchi, H. Molecular cloning, expression, and site-directed mutations of oxidosqualene cyclase from Cephalosporium caerulens. *Biochim. Biophys. Acta* **2001,** 1522, 67–73.
9. Hoshino, T.; Nakagawa, K.; Aiba, Y.; Itoh, D.; Nakada, C.; Masukawa, Y. Euphorbia tirucalli β-Amyrin Synthase: Critical Roles of Steric Sizes at Val483 and Met729 and the CH-pi Interaction between Val483 and Trp534 for Catalytic Action. *ChemBioChem* **2017,** 18, 2145–2155.
10. Ito, R.; Masukawa, Y.; Nakada, C.; Amari, K.; Nakano, C.; Hoshino, T. β-Amyrin synthase from Euphorbia tirucalli. Steric bulk, not the pi-electrons of Phe, at position 474 has a key role in affording the correct folding of the substrate to complete the normal polycyclization cascade. *Org. Biomol. Chem.* **2014,** 12, 3836–3846.
11. Ito, R.; Masukawa, Y.; Hoshino, T. Purification, kinetics, inhibitors and CD for recombinant β-amyrin synthase from Euphorbia tirucalli L and functional analysis of the DCTA motif, which is highly conserved among oxidosqualene cyclases. *Febs J.* **2013,** 280, 1267–1280.
12. Aiba, Y.; Watanabe, T.; Terasawa, Y.; Nakano, C.; Hoshino, T., Strictly Conserved Residues in Euphorbia tirucalli β-Amyrin Cyclase: Trp612 Stabilizes Transient Cation through Cation-pi Interaction and CH-pi Interaction of Tyr736 with Leu734 Confers Robust Local Protein Architecture. *ChemBioChem* **2018,** 19, 486–495.
13. Kushiro, T.; Shibuya, M.; Masuda, K.; Ebizuka, Y. Mutational Studies on Triterpene Synthases:

Engineering Lupeol Synthase into β-Amyrin Synthase. *J. Am. Chem. Soc.* **2000,** 122, 6816–6824.
14. Meyer, M.M.; Segura, M.J.; Wilson, W.K.; Matsuda, S.P. Oxidosqualene Cyclase Residues that Promote Formation of Cycloartenol, Lanosterol, and Parkeol. *Angew. Chem. Int. Ed. Engl.* **2000,** 39, 4090–4092.
15. Wu, T.K.; Liu, Y.T.; Chang, C.H.; Yu, M.T.; Wang, H.J. Site-saturated mutagenesis of histidine 234 of Saccharomyces cerevisiae oxidosqualene-lanosterol cyclase demonstrates dual functions in cyclization and rearrangement reactions. *J. Am. Chem. Soc.* **2006,** 128, 6414–6419.
16. Wu, T.K.; Wen, H.Y.; Chang, C.H.; Liu, Y.T. Protein plasticity: a single amino acid substitution in the Saccharomyces cerevisiae oxidosqualene-lanosterol cyclase generates protosta-13(17),24-dien-3beta-ol, a rearrangement product. *Org. Lett.* **2008,** 10, 2529–2532.
17. Liu, Y.T.; Hu, T.C.; Chang, C.H.; Shie, W.S.; Wu, T.K. Protein engineering of Saccharomyces cerevisiae oxidosqualene-lanosterol cyclase into parkeol synthase. *Org. Lett.* **2012,** 14, 5222–5225.
18. Wu, T.K.; Chang, Y.C.; Liu, Y.T.; Chang, C.H.; Wen, H.Y.; Li, W.H.; Shie, W.S. Mutation of isoleucine 705 of the oxidosqualene-lanosterol cyclase from Saccharomyces cerevisiae affects lanosterol's C/D-ring cyclization and 17alpha/beta-exocyclic side chain stereochemistry. *Org. Biomol. Chem.* **2011,** 9, 1092–1097.
19. Wu, T.K.; Li, W.H.; Chang, C.H.; Wen, H.Y.; Liu, Y.T.; Chang, Y.C. Differential Stereocontrolled Formation of Tricyclic Triterpenes by Mutation of Tyrosine 99 of the Oxidosqualene-Lanosterol Cyclase from Saccharomyces cerevisiae. *Eur. J. Org. Chem.* **2009,** 2009, 5731–5737.
20. Wu, T.K.; Chang, C.H. Enzymatic formation of multiple triterpenes by mutation of tyrosine 510 of the oxidosqualene-lanosterol cyclase from Saccharomyces cerevisiae. *ChemBioChem* **2004,** 5, 1712–1715.
21. Chang, C.H.; Chen, Y.C.; Tseng, S.W.; Liu, Y.T.; Wen, H.Y.; Li, W.H.; Huang, C.Y.; Ko, C.Y.; Wang, T.T.; Wu, T.K. The cysteine 703 to isoleucine or histidine mutation of the oxidosqualene-lanosterol cyclase from Saccharomyces cerevisiae generates an iridal-type triterpenoid. *Biochimie* **2012,** 94, 2376–2381.
22. Chang, C.H.; Wen, H.Y.; Shie, W.S.; Lu, C.T.; Li, M.E.; Liu, Y.T.; Li, W.H.; Wu, T.K. Protein engineering of oxidosqualene-lanosterol cyclase into triterpene monocyclase. *Org. Biomol. Chem.* **2013,** 11, 4214–4219.

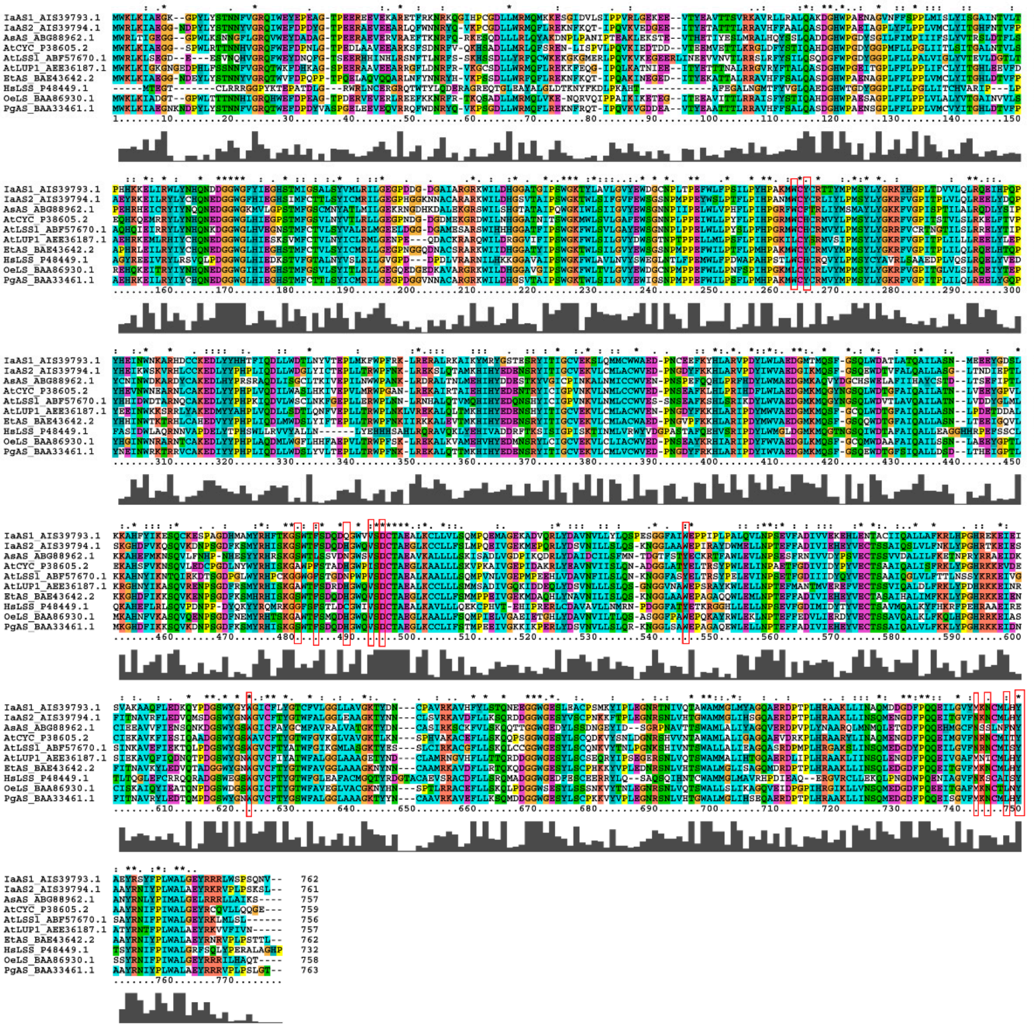

Supplementary Figure S1. Multi-sequence alignment of IaAS1, IaAS2 and other eight OSCs.

AsAS(ABG88962.1): beta-amyrin synthase of *Avena strigosa*, AtCYC(P38605.2): Cycloartenol synthase of *Arabidopsis thaliana*, AtLSSl(ABF57670.1): Lanosterol synthase of *Arabidopsis thaliana*, AtLUP1(AEE36187.1): Lupeol synthase 1 of *Arabidopsis thaliana*, EtAS(BAE43642.2): beta-amyrin synthase of *Euphorbia tirucalli*, HsLSS(P48449.1): Human Lanosterol synthase, OeLS(BAA86930.1): Lupeol synthase of *Olea europaea*, PgAS(BAA33461.1): beta-Amyrin Synthase of *Panax ginseng*. The reported site-directed mutations are marked in red boxes.
